# Optimal Exposure Biomarkers for Nonpersistent Chemicals in Environmental Epidemiology

**DOI:** 10.1289/ehp.1510041

**Published:** 2015-07-01

**Authors:** Antonia M. Calafat, Matthew P. Longnecker, Holger M. Koch, Shanna H. Swan, Russ Hauser, Lynn R. Goldman, Bruce P. Lanphear, Ruthann A. Rudel, Stephanie M. Engel, Susan L. Teitelbaum, Robin M. Whyatt, Mary S. Wolff

**Affiliations:** 1 Centers for Disease Control and Prevention, Atlanta, Georgia, USA; 2 National Institute of Environmental Health Sciences, National Institutes of Health, Department of Health and Human Services, Research Triangle Park, North Carolina, USA; 3 Institute for Prevention and Occupational Medicine of the German Social Accident Insurance, Ruhr-Universität Bochum, Bochum, Germany; 4 Icahn School of Medicine at Mount Sinai, New York, New York, USA; 5 Harvard T.H. Chan School of Public Health, Boston, Massachusetts, USA; 6 Milken Institute School of Public Health, George Washington University, Washington, DC, USA; 7 British Columbia Children’s Hospital, Vancouver, British Columbia, Canada; 8 Silent Spring Institute, Boston, Massachusetts, USA; 9 University of North Carolina at Chapel Hill, Chapel Hill, North Carolina, USA; 10 Mailman School of Public Health, Columbia University, New York, New York, USA

## Abstract

We discuss considerations that are essential when evaluating exposure to nonpersistent, semivolatile environmental chemicals such as phthalates and phenols (e.g., bisphenol A). A biomarker should be chosen to best represent usual personal exposures and not recent, adventitious, or extraneous exposures. Biomarkers should be selected to minimize contamination arising from collection, sampling, or analysis procedures. Pharmacokinetics should be considered; for example, nonpersistent, semivolatile chemicals are metabolized quickly, and urine is the compartment with the highest concentrations of metabolites. Because these chemicals are nonpersistent, knowledge of intraindividual reliability over the biologic window of interest is also required. In recent years researchers have increasingly used blood as a matrix for characterizing exposure to nonpersistent chemicals. However, the biologic and technical factors noted above strongly support urine as the optimal matrix for measuring nonpersistent, semivolatile, hydrophilic environmental agents.

Quantification of exposure biomarkers is increasingly used to provide an integrated measure of a person’s multiple chemical-specific exposures (Pirkle et al. 1995). Yet successful exposure characterization requires more than sophisticated analytical chemistry techniques—the biomarker and matrix are also key.

For general-population exposures, persistent organic pollutants (POPs) are usually measured in blood, and metabolites of nonpersistent chemicals such as phthalates and bisphenol A (BPA) are best measured in urine (Koch and Calafat 2009). In recent years, however, investigators have increasingly characterized exposure to nonpersistent chemicals by using other tissue matrices, particularly blood. To illustrate this trend, we identified 80 scientific articles, published in 2000–2014, that reported measuring BPA or phthalates in blood serum/plasma and other nonaqueous matrices, and that addressed topics on etiology, exposure, or metabolism (Figure 1).

Urine is the preferred matrix for most nonpersistent chemicals because of their pharmacokinetics. Nonpersistent chemicals are quickly transformed to hydrophilic, polar metabolites and excreted mainly in urine (Koch and Calafat 2009). The concentration of most urinary metabolites is 30–100 times greater than concentrations in blood (Engel and Wolff 2013; National Research Council 2008). With existing analytical techniques, higher urinary concentrations facilitate quantification, whereas relatively low blood concentrations of polar biomarkers increase the likelihood that external contamination obscures true exposures.

These are paramount considerations, given the increasingly recognized ubiquity of contamination. Controlled conditions of collection, storage, and processing of biospecimens are a long-acknowledged critical step for trace analyses of metals, volatile organic compounds, and POPs (Alcock et al. 1994; Ashley et al. 1992; Bolann et al. 2007). Unfortunately, the importance of preanalytic contamination sources is not as well understood for semivolatiles such as phthalates, phenols (e.g., BPA, parabens, triclosan), and similar modern nonpersistent chemicals.

Rather than reflecting a person’s usual exposure over months to years, detected biomarkers of chemicals such as phthalates and BPA can represent recent use of medical equipment or treatment near the time of biospecimen collection (Jaeger and Rubin 1970; Larson et al. 1977; Vandentorren et al. 2011; Yan et al. 2009). Extraneous contamination may occur during both the preanalytical and analytical phases. Phthalates and BPA can be detected even in the cleanest laboratories, from reagents, sampling equipment, and analytical apparatus (Fankhauser-Noti and Grob 2007; Longnecker et al. 2013; Marega et al. 2013; Markham et al. 2010; Vandenberg et al. 2014). Extraneous phenols may also come from sources such as plastics or paper products (BPA), soap (triclosan), moist towelettes (parabens), and preservatives (BPA, parabens) (Guidry et al. 2015; Longnecker et al. 2013; Ye et al. 2013). Moreover, in the field, during processing, or in the laboratory, beginning immediately after sample collection, phthalate diesters are hydrolyzed to monoesters by enzymes present in most biologic matrices (e.g., milk, serum, meconium, amniotic fluid, skin, saliva, sweat). These hydrolytic enzymes are not present in urine (Hines et al. 2009). In other words, monoesters are formed both from diesters absorbed in the body from a person’s general environment—the exposure we intend to assess—and from diesters introduced into the biospecimen itself from contaminated surfaces and medical devices.

Phase I (e.g., phthalate oxidative metabolites) and phase II (e.g., phenol conjugates) metabolite biomarkers are least likely to arise from extraneous sources (Koch and Calafat 2009). Also, these metabolites exist at higher levels than the hydrolytic monoesters or free (unconjugated) phenols in urine (Hines et al. 2009; Teeguarden et al. 2013). For many phenols, no specific oxidative metabolites that can exclude contamination are currently used for biomonitoring. Instead, conjugated phenols (rather than the unconjugated species) are the most valid exposure biomarkers (Koch et al. 2012; Teeguarden et al. 2013).

The short half-life of nonpersistent chemicals presents additional challenges, in that a biomarker must meet the criterion of temporality. Regardless of the matrix, intraindividual variability exists in exposure metrics over short time periods, particularly when such exposures are episodic in nature (Frederiksen et al. 2013; Koch et al. 2014; Lassen et al. 2013; Preau et al. 2010; Ye et al. 2011). However, metabolites are detectable longer in urine than in other matrices. Acceptable biomarker variability can exist because exposures arise from common, quotidian sources. Consequently, reasonable reproducibility over months to years has been found for urinary biomarker concentrations (Baird et al. 2010; Engel et al. 2014; Hauser et al. 2004; Mahalingaiah et al. 2008; Marcus et al. 2010; Meeker et al. 2012; Nepomnaschy et al. 2009; Teitelbaum et al. 2008; Townsend et al. 2013). To improve exposure assessment, studies can also incorporate pooling of specimens or repeated measurements across the time window of interest, such as trimesters of pregnancy (Adibi et al. 2008; Braun et al. 2011; Cantonwine et al. 2014; Fisher et al. 2014; Irvin et al. 2010; Jusko et al. 2014; Meeker et al. 2013; Philippat et al. 2013; Quirós-Alcalá et al. 2013; Smith et al. 2012; Valvi et al. 2015).

In population research, environmental agents are selected for study based on biological relevance and exposure prevalence. Exposure biomarkers should be based on pharmacokinetics of the target chemicals and a suitable sampling matrix. The design should incorporate appropriate control procedures for collecting and processing specimens, validated analytical methods, and statistical analyses that account for sampling issues such as urine dilution (Barr et al. 2005) and collection times (Calafat and Needham 2009).

Technology has made it analytically possible to measure polar biomarkers at trace concentrations in many media, but these endeavors risk exposure misclassification due to low biomarker concentrations, short biologic half-lives, and threat of external contamination. Greater reliance on phthalate and phenol biomarker concentrations in urine will improve the return on investment in environmental research.

**Figure 1:**
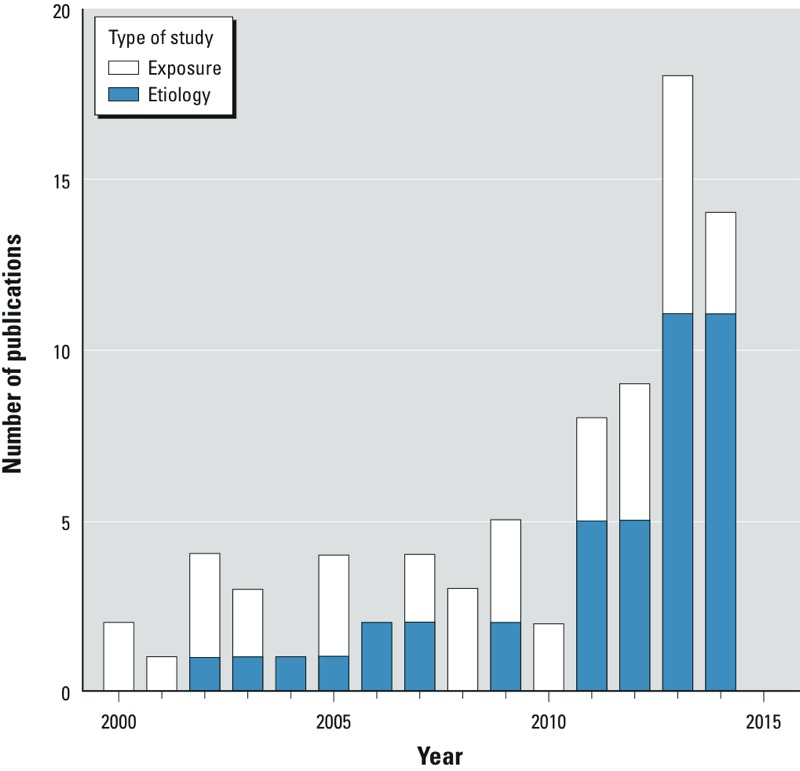
Number of publications per year using blood or other non-urine biomarkers for bisphenol A (BPA) or phthalates, by type of study, from 2000 through 2014 (n = 80). The search strategy aimed to capture studies with measurements of free BPA or phthalate made on a human matrix, published from 2000 through 2014. We excluded 16 papers that were exclusively about the analytical chemistry methods of assay. We searched for English-language articles using the terms “phthalate” or “BPA,” measured in human serum, plasma, semen, adipose, milk, or saliva. The following list of PubMed identifiers, pasted into PubMed, retrieves the 80 articles we assessed, of which 38 are etiologic and 42 are exposure assessment: 25371878[uid] or 25337790[uid] or 25296284[uid] or 25268510[uid] or 25227326[uid] or 25213476[uid] or 25048886[uid] or 25036990[uid] or 24974312[uid] or 24816463[uid] or 24724919[uid] or 24720399[uid] or 24550655[uid] or 24503621[uid] or 24378374[uid] or 24336026[uid] or 24255718[uid] or 24025997[uid] or 23941471[uid] or 23904340[uid] or 23761051[uid] or 23710608[uid] or 23710174[uid] or 23667484[uid] or 23651625[uid] or 23506159[uid] or 23441348[uid] or 23411151[uid] or 23404131[uid] or 23377699[uid] or 23347089[uid] or 23213291[uid] or 23145999[uid] or 24524038[uid] or 22805002[uid] or 22722103[uid] or 22578698[uid] or 22498808[uid] or 22402483[uid] or 22381621[uid] or 22267833[uid] or 22050967[uid] or 21875366[uid] or 21705716[uid] or 21527603[uid] or 21440837[uid] or 24278551[uid] or 22953188[uid] or 21193545[uid] or 20822678[uid] or 20579427[uid] or 19706995[uid] or 19555962[uid] or 19444800[uid] or 19426969[uid] or 19165392[uid] or 18577445[uid] or 18273031[uid] or 18245696[uid] or 17822133[uid] or 17689919[uid] or 17661831[uid] or 17049806[uid] or 16603434[uid] or 16451866[uid] or 15995852[uid] or 15947000[uid] or 15893743[uid] or 15847671[uid] or 15644579[uid] or 14594632[uid] or 12869118[uid] or 12566679[uid] or 12417499[uid] or 12407035[uid] or 12401500[uid] or 11829464[uid] or 11604266[uid] or 10964036[uid] or 10716589[uid].

The findings and conclusions in this report are those of the authors and do not necessarily represent the official position of the Centers for Disease Control and Prevention. This research was supported in part by the Intramural Research Program of the National Institute of Environmental Health Sciences (NIEHS), National Institutes of Health, Department of Health and Human Services. We thank Sandra Chambers at the NIEHS for help in identifying articles listed in Figure 1.
